# Observational Assessments of Chicken, Beef, and Seafood Proportions with a Mediterranean-Style Healthy Dietary Pattern and Cardiovascular Risk Factor Changes: Post Hoc Analysis of a Controlled Feeding Trial

**DOI:** 10.3390/nu18132062

**Published:** 2026-06-24

**Authors:** Eric M. Davis, Robert E. Bergia, Austin S. Hartman, Rikard Landberg, Gabriele Riccardi, Wayne W. Campbell

**Affiliations:** 1 Department of Nutrition Science, Purdue University, West Lafayette, IN 47907, USA; edavisresearch759@gmail.com; 2 Berkwood Research, Santa Barbara, CA 93109, USA; robbergia@gmail.com; 3 Department of Statistics, Purdue University, West Lafayette, IN 47907, USA; hartma91@purdue.edu; 4 Department of Biology and Biological Engineering, Food Science and Nutrition, Chalmers University of Technology, 41296 Gothenburg, Sweden; rikard.landberg@chalmers.se; 5 Diabetes, Nutrition and Metabolism Unit, Department of Clinical Medicine and Surgery, Federico II University, 80138 Naples, Italy; riccardi@unina.it

**Keywords:** Mediterranean diet, metabolic syndrome, cardiovascular disease risk factors, beef, chicken, salmon, shrimp, seafood, red meat, poultry

## Abstract

**Background**: We previously reported that consuming a Mediterranean-style healthy dietary pattern (MED-HDP) with lower vs. higher glycemic index foods differentially changed indices of postprandial glucose control and daily glycemic variability but did not influence improvements in cardiovascular health indices. **Methods**: Fifty-two adults (31 females, 21 males; aged 49 ± 11 y, BMI 31 ± 3.1 kg/m^2^, mean ± SD) with two or more features of metabolic syndrome participated for 12 weeks in the randomized, controlled trial with all foods provided. At dinner only, participants could select from protocol-approved foods, including unprocessed chicken breast, unprocessed lean beef, and unprocessed salmon and shrimp (seafood). **Objective**: Herein, we retrospectively assessed whether the frequency of consuming different sources of meat (i.e., the exposures) was associated with MED-HDP-induced changes in cardiovascular health indices (i.e., the outcomes). **Results**: Among all participants, consuming the MED-HDP foods (88% adherence) reduced fasting systolic (SBP) and diastolic (DBP) blood pressures and serum total cholesterol (TC), triglycerides (TGs), and HDL. More frequent consumption of chicken at dinner, in place of beef and seafood, was associated with greater reductions in SBP (*p* = 0.034 and *p* = 0.047 for replacing beef and seafood, respectively) and DBP (*p* = 0.021 and *p* = 0.043, respectively). Frequency of chicken, beef, and seafood intakes at dinner did not associate with the reductions in serum TC, TG, HDL, or LDL. **Conclusions**: These results support that adoption of a MED-HDP improved multiple cardiovascular risk factors among middle-aged and older adults at elevated cardiovascular risk. The observed modest associations between more frequent consumption of unprocessed chicken at dinner and greater blood pressure reductions, which do not mean that eating more chicken at dinner causes lower blood pressure, warrant independent replication.

## 1. Introduction

Dietary quality and healthfulness are associated with the prevention and management of cardiovascular diseases (CVDs). Consuming a Mediterranean-style Healthy Dietary Pattern (MED-HDP) is endorsed by leading health organizations, including the World Health Organization (WHO), American Heart Association (AHA), and American College of Cardiology (ACC), for its ability to promote cardiometabolic health [1,2]. A MED-HDP emphasizes consuming a variety of nutrient-dense foods, including vegetables, fruits, grains, dairy, protein foods, and oils [3]. For the 2020–2025 Dietary Guidelines for Americans [3], the “Protein Foods” food group of a MED-HDP include poultry, red meats, seafood, eggs, nuts, seeds, and soy products. There is an abundance of research assessing associations between the amounts of poultry, red meat, and seafood consumed and cardiovascular disease risk among groups of community-dwelling people who self-choose the foods they eat [4,5,6]. There is also experimental research with investigator-controlled feeding paradigms assessing the effects of consuming portioned amounts of poultry, red meat, or seafood on cardiovascular disease risk factors [7,8,9,10]. However, there seems to be a paucity of research assessing associations between the relative amounts of poultry, red meat, and seafood self-chosen by participants who are consuming a controlled MED-HDP with all foods provided.

Although MED-HDPs demonstrate profound cardiometabolic benefits across diverse populations [11,12], there is considerable heterogeneity in how the pattern is operationalized, particularly with respect to animal protein sources. Mediterranean regions vary substantially in meat consumption frequency and type [13], yet the overall dietary pattern has proven resilient across this variation. This heterogeneity raises a question relevant to both personalized nutrition and practical adherence: whether specific animal protein choices (e.g., poultry, beef, seafood) modulate the cardiovascular benefits of an otherwise well-defined MED-HDP. The MEDGI-Carb parent trial provides a unique opportunity to examine this question in a controlled feeding context where all food was provided and dietary pattern composition was standardized, allowing for isolation of meat-choice associations from overall dietary quality variation. Consistent with the health-promoting properties of a MED-HDP, results from the MEDGI-CARB controlled feeding trial included reductions in fasting systolic (SBP) and diastolic (DBP) blood pressures, serum total cholesterol (TC) and triglycerides (TGs) [14].

The objective of this retrospective, observational study is to assess whether the frequency with which participants chose to consume unprocessed chicken breast, lean beef, or seafood (salmon and shrimp) at dinner (i.e., the exposures) is associated with MED-HDP-induced improvements in CVD risk factors (i.e., the outcomes). The CVD risk factors assessed include changes in fasting SBP and DBP, and serum total cholesterol (TC), triglycerides (TG), high-density lipoprotein cholesterol (HDL), and low-density lipoprotein cholesterol (LDL). Because the parent trial was not designed to test meat-source effects, this analysis is exploratory. Conceptually, we frame the analysis around whether the cardiovascular benefits of a MED-HDP are sensitive to the frequency with which these different animal flesh foods are selected at dinner. It may be hypothesized that more frequent consumption of beef (a type of red meat), chicken (a type of poultry), and salmon and shrimp (types of seafood) may be associated with smaller, neutral, and greater reductions in these CVD risk factors, respectively.

## 2. Materials and Methods

This article presents findings from a post hoc analysis of data collected from the United States of America (USA) research site of the MEDGI-Carb trial. The MEDGI-Carb trial is an international multi-center randomized, controlled, parallel-group, 15-week trial including a 3-week baseline period followed by 12 weeks of controlled dietary intervention. The USA site was the Department of Nutrition Science, Purdue University, West Lafayette, Indiana. The study was initiated in January 2018 and the testing phase of the trial continued through December 2019. The study protocol was approved by the institutional review board at Purdue University: protocol 1501015662; approval 9 January 2018. The MEDGI-Carb trial is registered with the public trial registry Clinicaltrials.gov as NCT03410719; first posted 25 January 2018. Detailed descriptions of the MEDGI-Carb trial protocol are published [15]. Each participant signed a written consent form prior to starting the protocol and was provided with monetary compensation for their time.

### 2.1. Experimental Design

The original clinical trial was a parallel group, randomized, controlled trial, with the participants consuming a controlled, euenergetic, weight-maintenance diet for 12 weeks. The participants were instructed to consume the intervention-specific foods (all foods provided) to achieve a lower-glycemic or higher-glycemic MED-HDP (low-GI or high-GI, respectively). Participants were instructed to consume portioned amounts of all foods at each eating occasion. For breakfast, lunch, and snack eating occasions, the menus were strictly controlled. Pertinent to the current post hoc investigation, for dinner participants chose from a list of protocol foods, including the sources of meat (See Section 2.3. Dietary Control for more dietary information). Importantly, although the parent study was a randomized controlled trial, the exposure variable analyzed in this manuscript (frequency of meat choices at dinner) was not randomized. As such, the analysis and findings are observational. Results presented in this article are based on participant testing done at baseline and intervention week 12.

### 2.2. Eligibility Criteria

The eligibility criteria were established to select middle-aged and older adults at high risk for developing type 2 diabetes. Therefore, adults with a waist circumference > 102 cm (males) or >88 cm (females) and one additional trait of the metabolic syndrome, according to the National Cholesterol Education Program’s Adult Treatment Panel III [16], were recruited. The additional traits could include fasting, sitting SBP/DBP > 130/85 mmHg or taking medication to control high blood pressure; fasting plasma glucose 5.6–7.0 mmol/L; fasting serum TG 1.7–4.5 mmol/L; HDL < 1.0 mmol/L (males) or <1.3 mmol/L (females). Participants were required to maintain stable physical activity and medication use. Physical activity was assessed using the International Physical Activity Questionnaire at baseline and post-intervention. Medication use, including antihypertensive and lipid-lowering agents, was tracked throughout the trial; participants with stable medication regimens were permitted to continue without modification. Full details on inclusion criteria, recruitment, and consent procedures have been published [15].

### 2.3. Dietary Control

During the 3-week baseline period, all participants consumed their habitual, self-chosen, unrestricted diets. Throughout the 12-week intervention period, each participant was counseled to consume their assigned low-GI or high-GI MED-HDP. All foods and beverages (except water) were provided to the participants using an online grocery ordering system. Each participant procured the food weekly at a grocery store. They were instructed on how to store, prepare, portion, and document consumption of the protocol foods. We used a repeating seven-day rotation of daily menus throughout the dietary intervention period. Each daily menu included a combination of prescribed foods (breakfast, lunch, and snacks) and an item-specific version of the ‘Dinner Recipe Builder’ for dinner (Table 1). The Dinner Recipe Builder is a tool to promote self-efficacy by which participants were given the flexibility to mix and match meal foods/ingredients, while still following a MED-HDP. Inspiration for the Dinner Recipe Builder was derived from the Barilla Pasta Builder, which allows the user to customize the food/ingredients of their dinner based on preference of that evening while still maintaining a HDP and a consistent energy intake at each dinner.

The two group-specific sets of menus contained primarily the same foods and beverages, except for substitutions of major sources of carbohydrates in the meals. The low-GI diet emphasized carbohydrates from lower-GI foods, such as pasta, brown rice, and an assortment of breads such as all bran, rye, wheat, and flatbread. The high-GI menus emphasized carbohydrates from higher-GI foods, such as white potatoes, jasmine rice, wholegrain bread, and rusks. Other dietary components included avocado, eggs, Greek yogurt, olive oil, hummus, nuts, and a various assortment of fruits and vegetables. Each participant, independent of GI group assignment, was required to consume chicken twice per week, beef once per week, and seafood three times per week at non-dinner meals. For dinner, each participant chose from the following unprocessed animal Protein Foods options: chicken breast, beef sirloin, ground beef, salmon, and shrimp. Participants were able to choose either one protein for their dinner or mix multiple protein choices together. Dinner meat portion size was proportionate to each participant’s estimated energy requirement, and ranged from 168 g raw weight at 2100 kcal/d to 249 g raw weight at 3300 kcal/d.

Operationally, each participant corresponded via email weekly with a study staff member on what protocol foods they needed for the following week. All fresh, perishable foods were ordered weekly, while shelf-stable and frozen foods were ordered as needed. This ordering system allowed each participant to order the numbers of servings of chicken, beef, and seafood then wanted for the following week. Among the seven dinners per week, each participant self-chose which meat source to consume. There were no minimum or maximum servings per week of these meats required for dinner. When a participant consumed half-servings of two meat sources at the same meal, these quantities were noted in their daily food log and recorded as such on data spreadsheets.

Dietary adherence was assessed using data from participants’ self-reported consumption of protocol foods, substitutions, and/or additions of non-protocol foods. Two complementary mechanisms were used: (1) the Dinner Recipe Builder, where participants circled their daily selections from the prescribed menu options, and (2) weekly menu adherence checklists reviewed by study staff. Because the trial provided all dinner foods and participants selected only among the presented options, dietary exposure data were complete and objective, not recall-based. Detailed descriptions of the dietary protocol and controls are published [15].

### 2.4. Blood Pressure and Blood Lipid-Lipoprotein Measurements

All measurements were conducted during fasting morning clinic visits. Fasting, sitting SBP and DBP were measured in duplicate using an automated blood pressure cuff (BP785, HEM-7222-Z, Omron Healthcare, Inc., Kyoto, Japan) after 15 min of quiet rest, by trained study staff. If measurements differed by more than 5 mmHg, a third measurement was obtained. The mean of the measures was used for analysis. Blood compounds of interest for the current assessments include TC, TG, HDL, and LDL. Fasting blood samples were obtained from an antecubital vein and placed in tubes containing a clot activator to obtain serum. Serum tubes were held at room temperature for at least 15 min and then centrifuged at 4000× *g* at 4 °C for 15 min, and immediately refrigerated/kept on ice, processed, and aliquoted into microtubes. The aliquots were frozen at −20 °C within 2 h of sample collection, stored at this temperature for a maximum of one week, and then stored at −80 °C until thawed for analysis. All samples were analyzed at the end of the study to minimize batch effects. Descriptions of the blood pressure and serum lipid and lipoprotein measurements are published [15].

### 2.5. Statistical Analyses

The present analyses use distinct features of this controlled feeding trial, where individuals selected food choice at one eating occasion (dinner), within an otherwise controlled and prescribed pattern (e.g., breakfast, lunch, and snacks were prescribed, without choice). This nesting allows us to objectively capture meat selections within a standardized dietary pattern, negating many self-reporting issues that confound nutritional epidemiology work. Nonetheless, we acknowledge that causal inference remains limited and have repeatedly framed this as an exploratory, hypothesis-generating analysis throughout the manuscript.

The primary analysis followed an intention-to-treat plan. Covariates accounted for in all statistical models included age, sex, and BMI. Physical activity and medication use were assessed and monitored but were not included as statistical covariates; they were not expected to change substantially during a controlled feeding trial where energy intake was prescribed for weight maintenance. We acknowledge this as a limitation. Data from both high- and low-GI interventions were pooled to determine associations, with the statistical significance threshold set at *p* < 0.05. Baseline clinical measurement differences between sexes were calculated by means of a two-sample *t*-test. For each clinical measurement, baseline and post-intervention values were utilized in a linear regression change-model, with covariate adjustments for age, sex, and BMI to assess group × time interactions. The sample size was determined a priori based on power calculations for the parent trial’s primary hypothesis regarding glycemic effects of low- versus high-GI Mediterranean dietary patterns. This analysis was not pre-specified as a primary aim of the parent trial and should be interpreted as exploratory. As such, this analysis was not powered based on an independent a priori sample size calculation; therefore, the statistical power to detect associations between meat consumption frequency and cardiovascular outcomes may be limited.

Associations between dietary meat composition and changes in cardiovascular outcomes were assessed using multiple linear regression. The independent variables of primary interest were the dietary proportions of chicken and seafood (salmon and shrimp), each defined as the proportion of dinner consumed by a participant which contained some amount of that protein subcategory. Red meat (beef) served as an implicit reference category and was excluded from the model to avoid perfect multicollinearity; all contrasts involving red meat were therefore derived relative to this reference. For each cardiovascular outcome (e.g., systolic blood pressure, LDL cholesterol, etc.), a change score was computed as the dependent variable, defined as the difference between post-intervention and pre-intervention values. Sex, age, and BMI were included as covariates. All models were fit using ordinary least squares regression with the following form:

ΔY = β0 + β1(Chicken) + β2(Seafood) + β3(Sex) + β4(Age) + β5(BMI) + ϵ

Three pairwise contrasts were derived from the fitted model coefficients: chicken vs. red meat (B_1_), seafood vs. red meat (B_2_), and chicken vs. seafood (B_1_ − B_2_). The chicken vs. seafood contrast was computed as the linear combination β1 − β2, with its standard error derived from the full variance–covariance matrix to account for the correlation between estimates. Within each outcome, *p*-values for these three contrasts were adjusted using the single-step Tukey method. No correction was applied across outcomes, as each cardiovascular marker was treated as a pre-specified endpoint of interest. No interaction terms were included in the models. Because the three meat-type proportions sum to a constrained total, an increase in the dietary proportion of one meat type necessarily corresponds to a decrease in another; the regression coefficients for chicken and seafood therefore inherently represent substitution effects relative to red meat. The goal of this analysis was to estimate the overall direction and magnitude of these substitution associations across the intervention period, not to assess whether effects varied across levels of the covariates. Statistical analyses were conducted in R version 4.5.3 using the lm and multcomp packages (R Core Team, Vienna, Austria).

Power analyses were conducted to estimate the sample size required for an adequately powered future study. For each outcome, partial eta squared (η^2^) effect sizes were computed for the chicken and seafood regressors from the fitted regression models, representing the proportion of outcome variance uniquely attributable to each meat type after accounting for age, sex, and BMI. Partial η^2^ was converted to Cohen’s f^2^ (f^2^ = η^2^/(1 − η^2^)), which served as the effect size metric for sample size calculations using the pwr package in R. Required sample sizes were estimated for 80% power at a two-sided α = 0.05.

## 3. Results

### 3.1. Participant Baseline Characteristics

**Figure 1:**
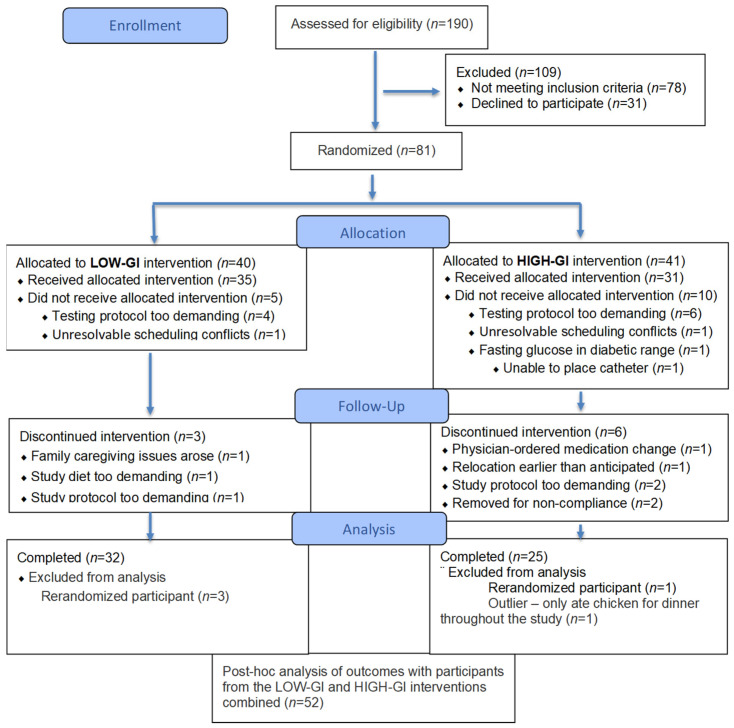
CONSORT flow diagram of study enrollment for the MEDGI-Carb USA center (LOW-GI and HIGH-GI, low-glycemic or high-glycemic Mediterranean-style healthy dietary pattern, respectively; *n*, sample size).

Eighty-one participants were consented and randomized into the study, with 66 starting and 57 completing the intervention, and data from 52 participants being used for assessments (Figure 1, CONSORT Flow Diagram). We studied 31 female and 21 male participants (Table 2), all presenting with elevated waist circumference, as stipulated by the inclusion criteria. The metabolic syndrome characteristics in our cohort varied, with 44% of the participants exhibiting two traits of the syndrome, 37% three traits (classified as having the metabolic syndrome), 16% four traits, and 3% all five traits. The most prevalent secondary trait was elevated blood pressure (60%), followed by high fasting glucose (46%), low HDL (38%), and high TG (33%).

### 3.2. Dietary Adherence of the MED-GI Carb Randomized Controlled Feeding Trial and Animal Protein Foods Selections at Dinner

Adherence to consuming protocol foods, including the self-chosen dinner meats, was approximately 88%. Among the animal protein foods, chicken breast strips were chosen for an average of 32.8% of the dinner meals, beef 42.3% (ground beef 23.9%, beef sirloin 18.4%), and seafood 24.9% (shrimp 13.1%, salmon 11.8%).

### 3.3. Overall Cardiovascular Health Responses to the MED-HDP and Justification for Combining Data from the Low-GI and High-GI MED-HDP Groups

Consumption of the high-GI MED-HDP versus the low-GI MED-HDP did not differentially affect intervention-induced changes in any of the outcomes of interest. The statistical group-by-time *p*-value for each parameter was >0.05 (range 0.39 to 0.87). Thus, data from all participants were used for these analyses.

Among all participants, consuming MED-HDP foods for 12 weeks reduced TC, TG, HDL, SBP, DBP, but not LDL (Table 3).

### 3.4. Associations Between Chicken, Beef, or Seafood Intakes and Changes in Lipids, Lipoproteins, and Blood Pressure

**Figure 2:**
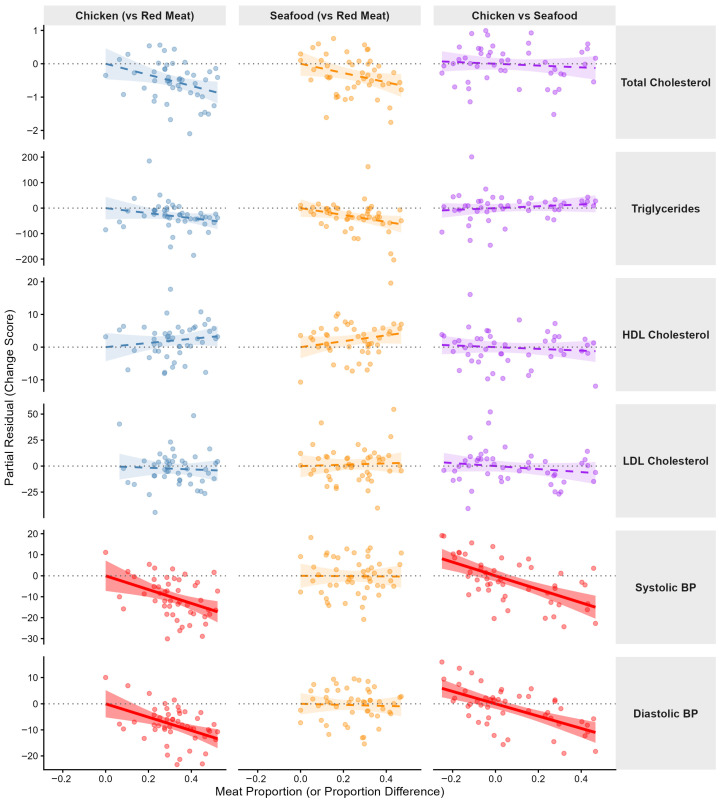
Partial-residual scatterplots of change in cardiovascular health indices against the proportion of dinner meals containing each meat type, adjusted for age, sex, and BMI. Solid red regression lines and shading indicate the contrasts with statistically significant associations after Tukey adjustment (*p* < 0.05); dashed lines indicate non-significant associations.

The proportion of dinner meals for which participants chose chicken, red meat, or seafood was not associated with changes in any of the lipid or lipoprotein parameters (Table 4). Greater frequency of choosing chicken instead of red meat or seafood was associated with greater reductions in SBP and DBP (Table 4 and Figure 2).

One participant chose to consume only chicken at dinner throughout the 12-week intervention period, which we deemed an outlier. To be sure this outlier’s data did not bias the findings, two analyses were done; one that included the outlier and one that did not. For SBP and DBP, these analyses support that excluding versus including this participant moderately influenced the associations. For SBP, partial η^2^ values were 0.108 (*p* = 0.034 *) and 0.092 (*p* = 0.036 *), respectively, for chicken vs. red meat contrasts and 0.026 (*p* = 0.998) and 0.029 (*p* > 0.999) respectively for seafood vs. red meat contrasts. For DBP, partial η^2^ values were 0.142 (*p* = 0.021 *) and 0.122 (*p* = 0.032 *), respectively, for chicken vs. red meat contrasts and 0.001 (*p* = 0.973) and 0.002 (*p* = 0.984) respectively for seafood vs. red meat contrasts. The ‘*’ identifies a significant association, *p* < 0.05; all *p*-values are Tukey-adjusted for multiple pairwise comparisons. Although the point estimates did not experience large deviations when excluding the outlier, because many of the significance levels sit near the rejection threshold, minor changes can result in different interpretations, especially in small-sample post hoc analyses. As such, a properly powered analysis of these potentially significant effects could provide a clearer insight. However, given the extreme nature of this participant’s diet relative to the rest of the study, we decided to remove this outlier from the analysis, as we did not consider them representative of the typical balanced diets seen in the rest of the study. We acknowledge that this exclusion serves as a potential limitation of this framework for investigating the effects of extreme diets with little to no variation from meal to meal.

Effect sizes for non-significant predictors were typically small by conventional benchmarks (partial η^2^ ≤ 0.06), though modest effects are expected in nutritional intervention research given the complexity of dietary influences on cardiovascular outcomes. Based on the observed effect sizes (Table 5), outcomes with moderate but non-significant effects (specifically change in TG and HDL associated with the seafood vs. red meat contrast, both with partial η^2^ ≥ 0.05) were associated with observed power ranging from 34.5% to 41.4% given the current sample of 52 participants, confirming the study may have been underpowered for several endpoints. For future studies assessing the influence of dietary meat substitutions on changes in cardiovascular disease risk factors after participants adopt a MED-HDP, the recommended sample sizes for 80% power to be able to detect all contrasts flagged as potentially moderate effect sizes are: TG = 125, HDL = 155, SBP = 71, and DBP = 54.

## 4. Discussion

The 2020–2025 Dietary Guidelines for Americans includes a recommendation to consume a MED-HDP to improve health [3]. Given the growing prevalence of cardiovascular and metabolic conditions and their associated health outcomes, it is important to understand how food components within a MED-HDP may influence overall pattern effectiveness. For example, results from our MEDGI-Carb randomized controlled trial support that greater consumption of low-glycemic index foods within a MED-HDP reduced postprandial and average daily plasma glucose concentrations, compared to baseline and compared to consuming a MED-HDP with higher-glycemic index foods [14,17]. For the current post hoc study, we assessed associations between how frequently participants chose to consume unprocessed chicken breast, lean beef, or seafood (salmon and shrimp) at dinner and MED-HDP-induced changes in CVD risk factors. The results suggest that choosing to consume chicken breast meat more frequently at dinner is associated with greater reductions in systolic and diastolic blood pressures. No associations were found between the frequency of consuming chicken and changes in lipids or lipoproteins, or the frequency of consuming beef or seafood at dinner and changes in any measured CVD risk factor.

Our finding that consuming a MED-HDP for 12 weeks reduced SBP and DBP is consistent with results from multiple randomized controlled trials (RCTs) chronicled in a systematic review and meta-analysis of dietary patterns and blood pressure in adults [18]. The observation from our post hoc analysis that more frequent consumption of unprocessed chicken breast for dinner (from rarely to about 50% of dinner meals) was associated with greater reductions in both SBP and DBP is interesting. Importantly, the experimental feature of allowing participants to choose how frequently they consumed chicken versus beef versus seafood for dinner within a MED-HDP is novel and precludes direct comparison with other diet-controlled RCTs where participants consumed specified amounts of various meats.

A paucity of experimental research exists on the effects of poultry (white meat) consumption and blood pressure [19], with limited RCTs with HDPs and fixed amounts of unprocessed chicken versus beef and (or) seafood published. Bergeron et al. [20] conducted an experiment with “generally healthy adults” that included two parallel dietary interventions (high and low saturated fatty acid intake) and within each intervention isonitrogenous amounts of red meat, white meat, or nonmeat protein foods (each consumed for 4 weeks). No significant effects of protein sources on blood pressure were observed. Using a randomized crossover experimental design, Sayer et al. [21] reported that among adults classified as older and overweight/obese, with elevated blood pressure, consuming a Dietary Approaches to Stop Hypertension (DASH) dietary pattern for six weeks reduced SBP and DBP. The participants consumed the DASH HDP twice (separated by a four-week dietary washout period), with chicken and fish versus lean pork as the predominant sources of protein (55% of total protein intake). The DASH-induced reductions in blood pressure were not influenced by these predominant sources of animal protein. The results of these experimental studies [20,21] are consistent with results from systematic reviews and meta-analyses of observational studies [22,23] suggesting the amount of white meat consumed may not be associated with blood pressure. This conclusion is based on data from people consuming their self-chosen diets with mixtures of processed and unprocessed white meats, which are typically less healthy than the MED-HDP with unprocessed chicken breast consumed by the participants in the current experimental study.

Dietary guidelines regarding beef intake for the United States are based primarily upon observational research [3]. Among experimental studies focusing on consumption of beef and cardiovascular risk factors, varied dietary patterns are used, thus adding further difficulty in understanding what causal effects beef has on cardiovascular health outcomes [7]. Conclusions from an Umbrella Systematic Review and Assessment of Causal Relations Using Bradford Hill’s Criteria included that while observational results suggest a weakly significant positive association between red meat and CVD risk factors, most experimental results do not support this relationship [5]. Based on results from a systematic review and meta-analysis of 20 randomized controlled trials, researchers concluded that “daily unprocessed beef intake do not significantly affect most blood lipids, apolipoproteins, or blood pressure, except for a small increase in LDL-cholesterol compared with diets with less or no beef [24].” Analysis of our associations mirrored their conclusions [5,7,24], as choice of beef for dinner was not associated with changes, either negative or positive, in any of our cardiovascular health outcomes when included in a MED-HDP. Plausible explanations for this may include: (1) lean unprocessed beef consumed as part of a MED-HDP does not associate with short-term changes in the measured CVD risk factors; (2) any negative influence of consuming red meat was negated by the overall healthfulness of the MED-HDP; and (3) other types of foods consumed with the MED-HDP (e.g., plant-based protein foods) also associate with CVD risk factor responses.

Observational results suggest that seafood, specifically fish, has an inverse correlation with the development of metabolic syndrome, along with positive cardioprotective benefits [6,25]. The current secondary analysis did not find any significant association between the frequency of seafood consumption and changes in cardiovascular health outcomes within a MED-HDP. There are a multitude of reasons why a discrepancy of conclusions exists between our findings and the aforementioned literature. One explanation involves the heterogeneity of fish-related interventions of the trials analyzed by these studies. Notably, these conclusions were based on data from multiple study designs, including observational, cohort, cross-sectional studies, and experimental trials. Trial interventions summarized by Widmer et al. [25] included both fish as a food product as well as fish oil in the form of supplementation, whereas our intervention only included fish as a food product. Therefore, dosage of beneficial nutrients in fish, such as omega fatty acids that have been shown to be protective of cardiovascular health, could be the reason for the discrepancy between our findings and those of the aforementioned studies.

One novelty of our experimental design was allowing each participant to choose which type of meat (and other menu items) to consume at dinner each evening within an otherwise prescribed MED-HDP. This model enabled participant preference for some dietary choices while maintaining a controlled feeding trial. The 88% dietary adherence during the 12-week period of dietary control is comparable to 92% dietary adherences during 8-week [26] and 12-week [27] controlled feeding interventions using the same grocery ordering and delivery system but without participants self-choosing some of their foods. These results support that high dietary adherence and flexibility within a controlled feeding study may be a favorable strategy for future experimental nutrition research trials [28].

This analysis has several methodological strengths. First, it leverages a controlled feeding trial design in which all food was provided, substantially reducing dietary measurement error and minimizing self-report bias that characterizes free-living dietary studies. As expected, among all participants, consumption of the MED-HDP reduced TC, TG, HDL, SBP, and DBP. Of note, the reduction in HDL, perhaps related to changes in dietary lipid and lipoprotein intakes and overall diet quality, is consistent with results from other short-term controlled feeding studies chronicled in meta-analyses [7,9]. We consider allowing participants to choose how often they consumed unprocessed beef, chicken, and seafood within a prescribed MED-HDP to be experimental strength. It is also noteworthy that in addition to their self-chosen meat sources at dinner, each participant was required to consume specific amounts (number of servings) of beef, chicken, and seafood weekly at non-dinner meals. Thus, the inverse associations between consuming chicken more frequently at dinner and reductions in SBP and DBP were not experimentally controlled or influenced by variable food intakes at non-dinner eating occasions. Second, meat exposure was quantified objectively through daily Dinner Recipe Builder documentation rather than retrospective food frequency questionnaires. Third, the inclusion of multiple distinct dinner meat options within a standardized MED-HDP provides a unique opportunity to examine meat-type associations independent of overall dietary quality variation.

Several limitations warrant consideration. This is a post hoc exploratory analysis. As such, these findings should be interpreted as hypothesis-generating rather than confirmatory. While the experimental design allowing participants to select what protocol-approved foods they consumed at dinner is vital to the current assessment, it precludes assessing dietary choice among the other eating occasions and daily food choices. Dinner meat selection was participant-driven and not randomly assigned, introducing potential confounding by unmeasured preferences or behavioral factors. This design also hindered comparison of other menu items that had prescribed amounts, such as cheeses and vegetables, from being compared in a similar manner. In addition, sample size was determined for the parent trial’s primary glycemic endpoints and may lack statistical power for the associations examined here. While providing 52 participants with all of their food for 12 weeks is a strength of this RCT to assess causation of the a priori independent variables on the dependent variables of interest, this cohort is relatively small when assessing the observational (non-causal) associations reported in this article. Finally, many of the observed correlations were modest in magnitude; this is expected in a controlled feeding trial where meat type represents a narrow exposure domain within an otherwise standardized diet.

This analysis was done with a food-centric perspective on meat selection patterns within a MED-HDP, rather than investigating specific nutrients or bioactive compounds in the meats. As such, our findings generate hypotheses about whole food associations that might motivate future mechanistically focused investigations. Future research focused on improving the quality of evidence, including a priori studies investigating variation in meats and other foods within MED-HDPs [29], along with unmeasured lifestyle and behavioral factors would help inform future dietary and public health recommendations for cardiovascular health.

## 5. Conclusions

Consistent with recommendations in the 2020–2025 Dietary Guidelines for Americans, we observed that middle-aged and older adults who have excess body weight and are at high risk for cardiometabolic diseases may improve their blood pressure, total cholesterol, and triglycerides by transitioning from their usual, less healthy diet to consuming a Mediterranean-style healthy diet. Retrospectively, we observed that greater frequency of unprocessed chicken consumption at dinner may be modestly associated with greater blood pressure reductions but not changes in lipids and lipoproteins. No associations were observed between the frequency of unprocessed lean beef or seafood consumption and changes in any of the cardiovascular outcomes measured. Importantly, these results are observational, should be replicated by new independent research, and do not mean that eating more chicken at dinner causes lower blood pressure.

## Figures and Tables

**Table 1:** Sample Dinner Recipe Builder with instructions to participants.

Dinner Recipe Builder					
Portion Size (In Grams)	Selection				
**Oil** **(9 g)**	Extra Virgin Olive Oil				
**Herbs** **(~1 g)**	Chives (chopped)	Italian Parsley	Oregano	Thyme	Basil
**Vegetable** **(1 cup)**	Tomato (coarse, chopped)	Asparagus; or Broccoli	Mushrooms	Zucchini	Spinach (fresh)
**Cheese** **(28 g)**	Asiago	Pecorino Romano	Mozzarella		
**Protein** **(168 g; raw)**	Ground Beef (95% lean)	Chicken Breast Strips	Lean Sirloin	Salmon	Shrimp
**Starch** **(83 g; dry)**	Pasta selection				
**Red wine (optional) (1–5 oz glass)**					
**Favorite Flavor**	Garlic (chopped)	Onion (chopped)	Celery (chopped)		

Instructions to participants: You are given the “Portion Size in Grams” to measure each of your ingredients. You may choose 1 item from each column to prepare your dinner: Circle which ingredients you use for your meal, including if you choose to have wine with your meal. 1. Pick your pasta and cook it for one minute less than package instructions. Drain reserving 1/2 cup of water. 2. Place your oil in a 12” skillet and warm over medium heat. 3. Add your flavor and gently cook until translucent. 4. Add your favorite protein and sauté on high heat until browned. 5. Add your choice of vegetables and continue to cook until wilted. 6. Stir in freshly cooked pasta and the 1/2 cup of reserved cooking water and cook for a few seconds until absorbed. 7. Remove from heat and add your choice of cheese. Mix and plate.

**Table 2:** Fasting clinical characteristics of participants at baseline.

Demographic Characteristics	All Participants (*n* = 52)	Male (*n* = 21)	Female (*n* = 31)	*p* Value *
Age, y	49 ± 11	49 ± 13	49 ± 10	
Female *n* (%)	31 (59%)			
Body Mass, kg	89.7 ± 13.8	94.6 ± 14.6	86.9 ± 12.8	0.061
BMI, kg/m^2^	31.1 ± 3.1	31.5 ± 3.1	31 ± 3.2	0.579
Waist Circumference, cm	107 ± 9.5	114 ± 7.3	103 ± 7.9	<0.001 *
Cardiometabolic				
Glucose, mg/dL	98 ± 8.8	100 ± 7.9	98 ± 10.4	0.327
TC, mg/dL	184 ± 37	179 ± 38	191 ± 39	0.321
TG, mg/dL	121 ± 58	138 ± 57	122 ± 84	0.417
HDL, mg/dL	46 ± 12	40 ± 11	50 ± 11	0.002 *
LDL, mg/dL	113 ± 32	110 ± 35	116 ± 30	0.531
SBP, mmHg	122 ± 12	126 ± 12	120 ± 13	0.101
DBP, mmHg	80 ± 7	79 ± 7	81 ± 7	0.331

Mean ± SD. * *p*-value < 0.05 at baseline, grouped by sex. TC: total cholesterol; TG: triglycerides; HDL: high-density lipoprotein cholesterol; LDL: low-density lipoprotein cholesterol; SBP: systolic blood pressure; DBP: diastolic blood pressure.

**Table 3:** Effects of consuming the Mediterranean-style healthy dietary pattern for 12 weeks on changes in cardiovascular disease risk factors.

Outcome	Pre	Post	Change	*p* Value
TC (*n* = 48)	186 ± 5.8	174 ± 5.7	−12 ± 2.6	<0.001 *
TG (*n* = 48)	129 ± 11.0	110 ± 10.6	−19 ± 7.9	0.020 *
HDL (*n* = 48)	45 ± 1.7	42 ± 1.4	−3 ± 0.9	0.001 *
LDL (*n* = 47)	114 ± 4.8	109 ± 4.4	−5 ± 2.5	0.054
SBP (*n* = 50)	122 ± 2	114 ± 2	−8 ± 2	<0.001 *
DBP (*n* = 50)	80 ± 1	75 ± 1	−5 ± 1	<0.001 *

Mean ± SEM. * *p* values < 0.05 are for the change over time (Δ); TC: total cholesterol (mg/dL); TG: triglycerides (mg/dL); HDL: high-density lipoprotein cholesterol (mg/dL); LDL: low-density lipoprotein cholesterol (mg/dL), SBP: systolic blood pressure (mmHg); DBP: diastolic blood pressure (mmHg).

**Table 4:** Contrasts between percentage of protein food choice at dinner and changes in cardiovascular risk factors.

Outcome	Contrast	Estimate ± SE	95% Confidence Interval	*p*-Value * Tukey-Adjusted
TC	Chicken vs. Red Meat	−1.7 ± 0.8	−3.6, 0.3	0.118
	Seafood vs. Red Meat	−1.4 ± 0.8	−3.4, 0.6	0.212
	Chicken vs. Seafood	−0.3 ± 0.8	−2.3, 1.8	0.943
TG	Chicken vs. Red Meat	−98.7 ± 77.6	−287.4, 90.0	0.419
	Seafood vs. Red Meat	−134.7 ± 76.7	−321.2, 51.8	0.197
	Chicken vs. Seafood	36.0 ± 79.5	−157.3, 229.4	0.893
HDL	Chicken vs. Red Meat	6.4 ± 7.7	−12.2, 25.0	0.686
	Seafood vs. Red Meat	9.0 ± 7.6	−9.4, 27.4	0.464
	Chicken vs. Seafood	−2.7 ± 7.8	−21.7, 16.4	0.939
LDL	Chicken vs. Red Meat	−8.0 ± 27.2	−74.1, 58.1	0.953
	Seafood vs. Red Meat	6.5 ± 24.3	−52.7, 65.6	0.962
	Chicken vs. Seafood	−14.5 ± 26.3	−78.3, 49.4	0.847
SBP	Chicken vs. Red Meat	−32.9 ± 12.7	−63.7, −2.1	0.034 *
	Seafood vs. Red Meat	−0.7 ± 12.7	−31.5, 30.0	0.998
	Chicken vs. Seafood	−32.2 ± 13.1	−63.9, −0.4	0.047 *
DBP	Chicken vs. Red Meat	−25.6 ± 9.2	−47.9, −3.4	0.021 *
	Seafood vs. Red Meat	−2.1 ± 9.2	−24.3, 20.2	0.973
	Chicken vs. Seafood	−23.6 ± 9.5	−46.5, −0.6	0.043 *

All values adjusted for sex, age, and BMI. * *p* values < 0.05. TC: total cholesterol; TG: triglycerides; HDL: high-density lipoprotein cholesterol; LDL: low-density lipoprotein cholesterol; SBP: systolic blood pressure; DBP: diastolic blood pressure.

**Table 5:** Effect sizes for contrasts between percentage of protein food choice at dinner and changes in cardiovascular risk factors.

Outcome	Predictor	Partial η^2^	Observed Power	Sample Size for 80% Power
TC	Chicken vs. Red Meat	0.026	0.196	305
	Seafood vs. Red Meat	0.047	0.325	166
	Chicken vs. Seafood	0.003 *	0.064	3024
TG	Chicken vs. Red Meat	0.006	0.081	1361
	Seafood vs. Red Meat	0.062	0.414	125
	Chicken vs. Seafood	0.005 *	0.077	1576
HDL	Chicken vs. Red Meat	0.001	0.058	5294
	Seafood vs. Red Meat	0.05	0.345	155
	Chicken vs. Seafood	0.003 *	0.065	2804
LDL	Chicken vs. Red Meat	<0.001	0.051	29,017
	Seafood vs. Red Meat	0.007	0.087	1159
	Chicken vs. Seafood	0.008 *	0.091	1043
SBP	Chicken vs. Red Meat	0.108	0.657	71
	Seafood vs. Red Meat	0.026	0.2	297
	Chicken vs. Seafood	0.123 *	0.719	62
DBP	Chicken vs. Red Meat	0.142	0.788	54
	Seafood vs. Red Meat	0.001	0.056	6525
	Chicken vs. Seafood	0.126 *	0.732	61

* Partial η^2^ for the chicken vs. seafood contrasts were derived from the contrast t-statistic as t^2^/(t^2^ + *df*_residual_), rather than directly from the SS decomposition of the model, as this comparison is a derived linear combination of model coefficients rather than an independent predictor.

## Data Availability

The data used for this study are available upon request from the corresponding author.
